# Non-puerperal uterine inversion due to submucous myoma in a young woman: a case report

**DOI:** 10.1186/1752-1947-4-21

**Published:** 2010-01-24

**Authors:** Marjolijn de Vries, Denise Arlette Maria Perquin

**Affiliations:** 1 Department of Obstetrics and Gynaecology, Medical Center Leeuwarden, 8901 BR Leeuwarden, the Netherlands

## Abstract

**Introduction:**

Inversion of the uterus is an uncommon complication of the puerperium and it is an even rarer complication of the non-puerperal period. A submucous myoma is mostly the cause of the non-puerperal inversion but diagnosis can be difficult. In young women, non-puerperal uterine inversion is likely associated with a malignancy.

**Case presentation:**

A 19-year-old nulliparous woman presented with abnormal vaginal bleeding, dysmenorrhoea, and a large mass protruding from her cervix. The mass was interpreted as a prolapsed pedunculated submucosal myoma. After extirpation of the mass by clamping and twisting its pedicle, a laparotomy was required under suspicion of a uterine rupture. The diagnosis was confirmed and the patient's uterus could be preserved. Pathological examination revealed a submucous myoma. The uterine inversion happened when the uterus retracted to expel the submucous myoma with fundal attachment. By extirpating the stalk the fundus was also resected, causing a uterine rupture.

**Conclusion:**

We report a case of non-puerperal uterine inversion associated with a benign submucous myoma. Non-puerperal uterine inversion is very uncommon in women of reproductive age and is usually caused by a malignant tumour. However, uterine-sparing surgery should be attempted in young women until the final pathology is known.

## Introduction

Uterine inversion is a rare complication of delivery. A non-puerperal uterine inversion is even more uncommon, with only 150 cases published between 1887 and 2006 [1,2]. The diagnosis can be difficult even on physical examination. Most non-puerperal inversions are caused by benign submucous myomas, while other causes are leiomyosarcoma, rhabdomyosarcoma, malignant mixed müllerian tumour and endometrial polyp. Women of reproductive age who present with the rare finding of non-puerperal uterine inversion are likely to have a malignancy. We report a case of non-puerperal uterine inversion due to a benign submucous myoma in a woman.

## Case presentation

A 19-year-old nulliparous woman presented with abnormal vaginal bleeding and dysmenorrhoea. Her medical history was uneventful. Pelvic examination revealed the presence of a large mass measuring 5 cm in diameter and protruding from the cervix. The preoperative ultrasound demonstrated a uterus with heterogeneous material visualized within a thickened endometrial cavity measuring 13.9 × 21.4 mm. Laboratory tests showed low haemoglobin level of 5.2 g/dL. With these findings we interpreted the mass as a prolapsed pedunculated submucous myoma. A hysteroscopic resection of the myoma was planned.

Two weeks after the outpatient visit, an examination under anesthesia revealed a remarkable increase in the degree of the prolapse of the large mass. The mass was noted to extend outside the vagina and beyond a palpable dilated cervix (instead of outside the cervix, which was the case on presentation). The myoma was clamped and twisted around its pedicle and the stump was ligated under direct vision. Excess downward traction on the myoma was avoided in order not to cause an inversion of the uterus. Electrocoagulation was performed when bleeding from the pedicle stump persisted. After the procedure the fundus was impalpable and the cervix could not be visualized on pelvic examination. The bladder was empty so it was not possible to misinterpret it as the uterus. After this, a fimbria appeared in the vagina. These findings made us strongly consider the possibility of a uterine rupture. A laparoscopy followed by laparotomy with Pfannenstiel incision was performed and the fundus of the uterus was perforated from the left cornual area to the right. The uterus could be preserved and was closed in layers. The postoperative period was uneventful and the patient was discharged from the hospital five days after the surgery. At follow-up her menstrual cycle was regular without dysmenorrhoea. The final histopathological report revealed a benign submucous leiomyoma.

The uterine inversion happened when the uterus retracted to expel the submucous myoma with fundal attachment. By extirpating the stalk the fundus was also resected, thus causing a uterine perforation.

## Discussion

There are two types of uterine inversions: puerperal or obstetric and non-puerperal or gynaecologic [3]. A uterine inversion is a rare complication of the puerperium and a non-puerperal inversion is an extremely rare occurrence. Gomez-Lobo *et al. *[1] reported 150 cases of non-puerperal uterine inversions documented from 1887 to 2006. In general, non-puerperal uterine inversion presents after 45 years and is mostly related to benign myomas and seldom associated with malignancies. Malignancies are commonly caused by leiomyosarcomas, or less commonly by endometrial carcinoma, rhabdomyosarcoma, malignant mixed müllerian tumour or endometrial stromal sarcoma.

Only four cases of non-puerperal uterine inversions have been reported in women aged less than 45 years by Gomez-Lobo *et al. *[1]. In addition, four cases in young adults were noted. Three women, aged 15, 16 and 18 years, were diagnosed with rhabdomyosarcoma [4-6] and Ueda *et al*. [7] reported a uterine inversion in a 28-year-old diagnosed with endometrial cancer. These case reports support the notion that a non-puerperal uterine inversion in women of reproductive age is associated with malignancy. Our case report is the only one that mentions an inversion that was associated with a benign submucous myoma.

The aetiology of uterine inversion is not clearly defined. Possible explanations could be a thin uterine wall, rapid growth of the tumour, tumour size, fundic localisation of the tumour, tumour attachment to the uterine wall with a thin pedicle, dilatation of the cervix by distension of the uterine cavity, and sudden expulsion of the tumour [2,8].

The main symptoms of non-puerperal uterine inversions are anaemia caused by irregular vaginal bleeding, vaginal discharge, lower abdominal and/or pelvic pain, a protruding mass in the vagina, and in some cases obstruction of the urethra.

Our case report confirms that the diagnosis of inversion may be difficult to make during examination. We note two determining findings, namely: (1) the uterine corpus is not palpated on bimanual examination, and (2) the cervix cannot be visualised after the vaginal mass was excised [9].

Imaging procedures such as ultrasound and magnetic resonance imaging will contribute to the diagnosis [10]. Unfortunately, because of the rare nature of the disorder, uterine inversion frequently goes undetected until surgery unless a high index of suspicion is maintained.

## Conclusion

We report a case of non-puerperal uterine inversion due to benign submucous myoma in a young woman. Non-puerperal uterine inversion is very uncommon in women of reproductive age and is usually caused by a malignant tumour. Diagnosis is difficult and often obtained during operation but radiological evaluation may help in making the correct diagnosis preoperatively. Uterine-sparing surgery should be attempted in young women until the final pathology of the disorder is known.

## Competing interests

The authors declare that they have no competing interests.

## Consent

Written informed consent was obtained from the patient for publication of this case report and any accompanying images. A copy of the written consent is available for review by the Editor-in-Chief of this journal.

## Authors' contributions

MV and DP were involved in patient care. MV collected the background data and wrote the manuscript. DAMP was involved in drafting the manuscript and providing feedback on earlier drafts. All authors read and approved the final manuscript.
